# Prevalence and Risk Factors Associated with *Entamoeba histolytica/dispar/moshkovskii* Infection among Three Orang Asli Ethnic Groups in Malaysia

**DOI:** 10.1371/journal.pone.0048165

**Published:** 2012-10-25

**Authors:** Tengku Shahrul Anuar, Hesham M. Al-Mekhlafi, Mohamed Kamel Abdul Ghani, Emelia Osman, Azlin Mohd Yasin, Anisah Nordin, Siti Nor Azreen, Fatmah Md Salleh, Nuraffini Ghazali, Mekadina Bernadus, Norhayati Moktar

**Affiliations:** 1 Department of Parasitology and Medical Entomology, Faculty of Medicine, Universiti Kebangsaan Malaysia, Kuala Lumpur, Malaysia; 2 Department of Parasitology, Faculty of Medicine, University of Malaya, Kuala Lumpur, Malaysia; 3 Programme of Biomedical Sciences, School of Diagnostic and Applied Health Sciences, Universiti Kebangsaan Malaysia, Kuala Lumpur, Malaysia; Université Pierre et Marie Curie, France

## Abstract

**Background:**

*Entamoeba histolytica/Entamoeba dispar/Entamoeba moshkovskii* infection is still prevalent in rural Malaysia especially among Orang Asli communities. Currently, information on prevalence of this infection among different ethnic groups of Orang Asli is unavailable in Malaysia. To contribute to a better comprehension of the epidemiology of this infection, a cross-sectional study aimed at providing the first documented data on the prevalence and risk factors associated with *E. histolytica/E. dispar/E. moshkovskii* infection was carried out among three Orang Asli ethnic groups (Proto-Malay, Negrito, and Senoi) in selected villages in Negeri Sembilan, Perak, and Pahang states, Malaysia.

**Methods/Findings:**

Faecal samples were examined by formalin-ether sedimentation and trichrome staining techniques. Of 500 individuals, 8.7% (13/150) of Proto-Malay, 29.5% (41/139) of Negrito, and 18.5% (39/211) of Senoi were positive for *E. histolytica/E. dispar/E. moshkovskii*, respectively. The prevalence of this infection showed an age-dependency relationship, with higher rates observed among those aged less than 15 years in all ethnic groups studied. Multivariate analysis confirmed that not washing hands after playing with soils or gardening and presence of other family members infected with *E. histolytica/E. dispar/E. moshkovskii* were significant risk factors of infection among all ethnic groups. However, eating with hands, the consumption of raw vegetables, and close contact with domestic animals were identified as significant risk factors in Senoi.

**Conclusions:**

Essentially, the findings highlighted that *E. histolytica/E. dispar/E. moshkovskii* parasites are still prevalent in Malaysia. Further studies using molecular approaches to distinguish the morphologically identical species of pathogenic, *E. histolytica* from the non-pathogenic, *E. dispar* and *E. moshkovskii* are needed. The establishment of such data will be beneficial for the public health authorities in the planning and implementation of specific prevention and control strategies of this infection in different Orang Asli ethnic groups in Malaysia.

## Introduction

Amoebiasis, an infection by the protozoan parasite *E. histolytica* is globally considered as a leading parasitic cause of human mortality besides malaria and schistosomiasis [1]. It is estimated that *E. histolytica* may infect half a billion people annually, with 100,000 deaths worldwide [2]. In developed countries, amoebiasis tends to be more common in older individuals and occurs mostly among homosexual men or in institutions [3]. However, in tropical regions, the epidemiology of amoebiasis is completely different and is more common among the general population and particularly among patients attending health care centres with diarrhoea [4]. Clinical features of amoebiasis range from asymptomatic colonization to amoebic dysentery and invasive extraintestinal amoebiasis which is manifested most commonly in the form of liver abscess. Out of 10% of the world’s population infected by *E. histolytica*, only 1% develops invasive form of the disease [1].

In Malaysia, food and water-borne diseases which are closely associated with environmental and personal hygiene practices are still among the major health problems especially among Orang Asli communities; Malaysian aborigines comprising 0.6% of the total population. The prevalence of *E. histolytica/E. dispar/E. moshkovskii* in Malaysia ranges from 1% to 61% [5]. To the best of our knowledge, there is a paucity of available documented data describing the prevalence and risk factors for *E. histolytica/E. dispar/E. moshkovskii* in the Proto-Malay and Negrito that can be used for comparison with Senoi ethnic group.

The present study was carried out in three Orang Asli ethnic groups (Proto-Malay, Negrito, and Senoi) in Malaysia. Using a cross-sectional design, the purpose of this study was to assess the prevalence and to identify underlying risk factors associated with *E. histolytica/E. dispar/E. moshkovskii* infection among these ethnic groups.

## Materials and Methods

### Study Area

**Figure 1:**
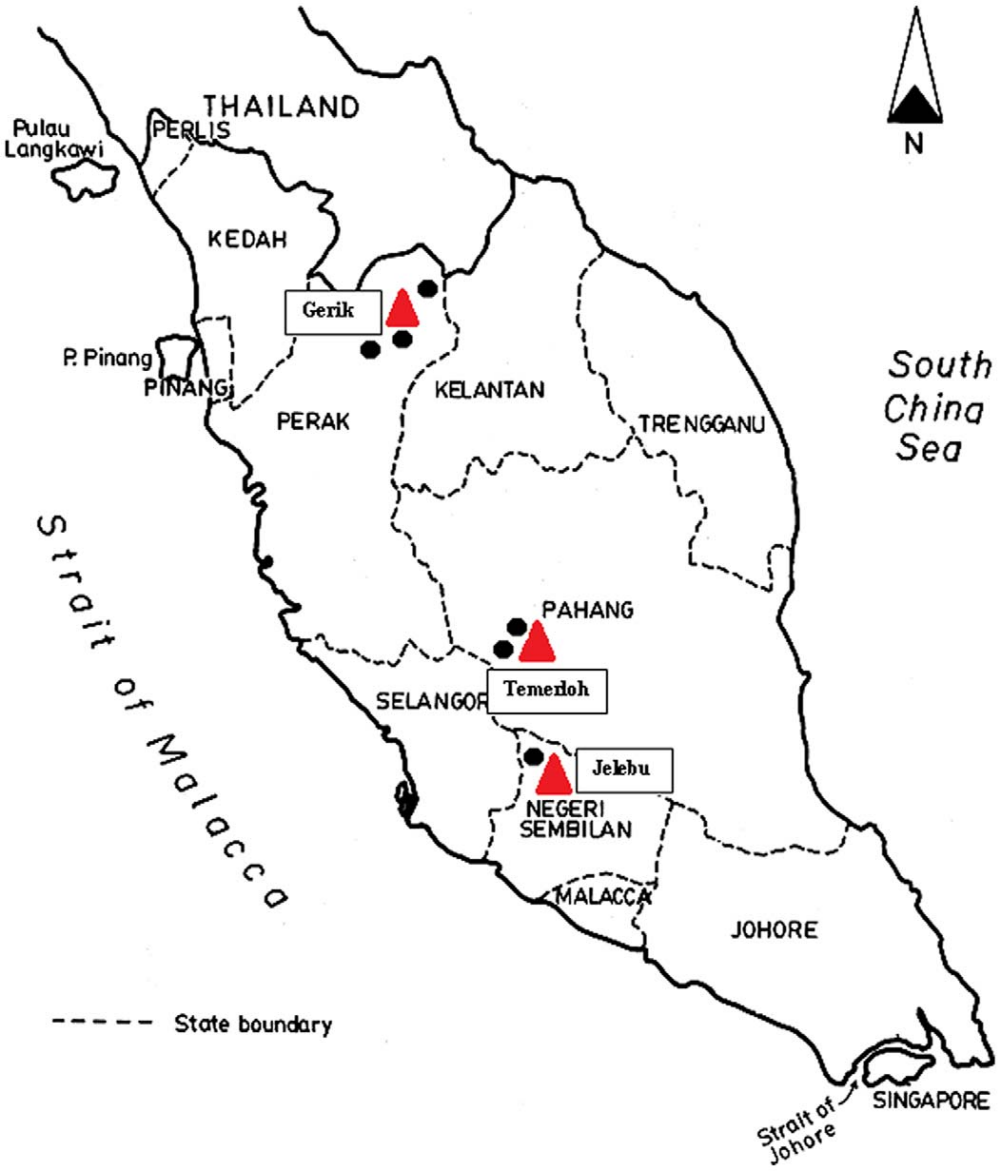
Map showing the location of the villages in Peninsular Malaysia involved in the study (triangles).

The present study was carried out from June to December 2011 in three different states of Peninsular Malaysia without discriminating age or gender. Specific villages within the locations of Jelebu (2° 55′ N latitude, 102° 4′ E longitude), Gerik (5° 26′ N latitude, 101° 7′ E longitude), and Temerloh (3° 43′ N latitude, 102° 22′ E longitude) in Malaysia were included in this study (Figure 1). These three states have a similar climate which is a tropical rainforest climate, being hot, and humid throughout the year.

Parit Gong village, Jelebu, Negeri Sembilan state is considered a sub-urban area with a population of 496 inhabitants. Most of the residents are primarily involved in farming and rubber tapping. There are also many individuals who are engaged in commercial and professional activities such as teaching and government service [6]. The average temperature in this area is 26.6°C whereby the average rainfall is 142 mm/year [7]. The main sub-ethnic residing in this area is the Temuan and they belong to the Proto-Malay ethnic group.

RPS Air Banun area, Gerik, Perak state is considered a remote area, located in a valley approximately 40 kilometres from the town of Gerik. The occupations of the inhabitants include farmers, rubber tappers, and some do odd jobs such as selling forest products. The area comprises five villages with 10–15 households in each [8]. The average temperature in this area is 26.9°C whereby the average rainfall is 180 mm/year [7]. The main sub-ethnic residing in this area is the Jahai which belongs to the Negrito ethnic group.

Pasu village, Temerloh, Pahang state is considered a sub-urban area which is within 10 kilometres from the town of Kuala Krau. Out of 625 residents, 65% are farmers and rubber tappers while the remainders are mostly government and private workers [9]. The average temperature in this area is 25.9°C whereby the average rainfall is 161 mm/year [7]. The main sub-ethnic residing in this area is the Jahut and they belong to the Senoi ethnic group.

### Sample Size

By using formula provided by Kish [10], the expected sample size was calculated according to the following parameters; expected prevalence of *E. histolytica/E. dispar/E. moshkvoskii* at 10% and 20% [11], [12], confidence interval at 95% and absolute precision (d) = 0.05 [13]. The required minimum sample size needed in this study was 138 individuals and maximum would be 246 individuals for each ethnic group.

### Study Design and Population Surveyed

Our cross-sectional surveys were carried out between June and December 2011. In each ethnic group, one to three villages were selected from the available village list in collaboration with the JAKOA, and 15–20 households were randomly selected in each village. All household members aged ≥2 years were invited to participate. The number of inhabitants per household was recorded. Unique identifiers were assigned to households and study participants.

### Questionnaire Survey

A structured questionnaire was developed in English and then translated to Malay language (the national language for Malaysia). The questionnaire was pre-tested among Orang Asli individuals who were admitted to Gombak Hospital, Selangor state. Trained research assistants interviewed participants in person, asking questions on demographic data (i.e., age, gender, and education level), socioeconomic background (i.e., occupation, household income, and educational status), behavioural risks (i.e., personal hygiene such as hand washing and food consumption), environmental sanitation and living condition characteristics (i.e., types of water supply, latrine system, sewage disposal system, and presence of domestic animals). Participants were also asked if they had diarrhoea and symptoms of gastroenteritis (i.e., vomiting, nausea, abdominal pain, watery stools and blood or mucus stools). For children, the questionnaire was completed by interviewing their parents or the guardian who had given informed consent.

### Parasitological Survey: Field and Laboratory Procedures

Following the administration of the questionnaire, a wide mouth screw-capped container pre-labelled with the individual’s name and code was distributed to each participant for the collection of a faecal sample the next day. Their ability to recognize their name was counter-checked. The participant was instructed to scoop a thumb sized faecal sample using a provided scoop into the container. Then, the container was placed into a zip-locked plastic bag. Parents and guardians were instructed to monitor their children during the sample collection in order to ensure that they placed their faecal samples into the correct container. All study participants were asked to provide sufficiently large faecal sample (at least 10 grams) so that both formalin-ether sedimentation and trichrome staining techniques could be performed. This study had to rely on a single faecal sample collection because of the limitation of resources and the cultural belief of the aborigines against giving of their faecal samples.

Faecal samples were processed in the designated area of work in the study village within a maximum of six hours after collection by experienced laboratory technicians. Approximately 5 grams of each faecal sample was kept into a 15 mL centrifuge tube containing 3 mL Polyvinyl Alcohol (PVA). PVA-fixed samples were forwarded to the parasitological department of the Faculty of Medicine, Universiti Kebangsaan Malaysia. The samples were subjected to trichrome staining. Briefly, the smear cover slip was stained as follows: iodine alcohol (15 minutes), 70% alcohol (10 minutes), trichrome stain (10 minutes), acid alcohol (3 seconds), 95% alcohol (5 minutes), absolute alcohol (5 minutes), and winter green oil (5 minutes) [14]. The cover slip was mounted using DPX and examined under light microscope at magnifications of 1000×. Additionally, another half of the samples were kept unfixed and stored at 4°C upon arrival at the laboratory for further analysis using formalin-ether sedimentation. Briefly, 2 grams of faecal sample was mixed with 7 mL of formalin and 3 mL of ether, centrifuged, stained with Lugol’s iodine, and finally examined under light microscope at magnifications of 400× [15]. Sample was reported as positive if cysts and/or trophozoites were detected by any of the two techniques.

Morphological characteristics of trophozoites and cysts (one to four spherical vesicular nuclei each containing a central karyosome, nuclear membrane lined with a thin layer of chromatin, presence of choromatoidal bars in cytoplasm, and measurement ranging from 10 to 20 µm) were used to identify *E. histolytica/E. dispar/E. moshkovskii* by microscopy [16].

### Data Analysis

Data was entered in a Microsoft Access and was cross-checked by the technical staff in order to ensure that data were entered correctly. Statistical analysis was performed using the SPSS version 20 (SPSS, Chicago, IL, USA). Only those individuals who had formalin-ether sedimentation and trichrome staining results together with complete questionnaire data were included in the final analyses.

For descriptive analysis, rate (percentage) was used to describe the characteristics of the studied population, including the prevalence of *E. histolytica/E. dispar/E. moshkovskii*. A Chi-squares test (χ^2^) was used to test the associations between the variables. In the univariate analysis, the dependent variable was prevalence of *E. histolytica/E. dispar/E. moshkovskii*, while the independent variables were demographic and socioeconomic factors, behaviour risks, environmental sanitation, living condition characteristics, and gastrointestinal symptoms. All variables that were significantly associated with the prevalence of *E. histolytica/E. dispar/E. moshkovskii* in the univariate model were included in a logistic regression analysis to identify the risk factors for *E. histolytica/E. dispar/E. moshkovskii* infection. For each statistically significant factor, an Odds Ratio (OR) and 95% confidence interval (CI) were computed by the univariate and multivariate logistic regression analyses. The level of statistical significance was set as *P*<0.05.

### Ethical Consideration and Treatment

Prior to data collection, the study protocol (Reference Number: UKM 1.5.3.5/244/FF-165-2011) was reviewed and approved by the Ethics Committee of Universiti Kebangsaan Malaysia Medical Centre (UKMMC) and permission for field work was obtained from Department of Orang Asli Development (JAKOA). Village meeting were held and village authorities and villagers were handed detailed explanations of the aims, procedures, potential risks, and benefits of the study. During the meeting, they were also informed that their identity and personal information would be kept strictly confidential, and they could withdraw from the study at any point of time without citing reasons for doing so. If they agreed to participate, their consent was obtained in written form (signature or thumbprint for those who were illiterate) or parents were approached for consent on behalf of their children.

Since molecular methods were not applied in this study to differentiate the pathogenic (*E. histolytica*) and non-pathogenic (*E. dispar* and *E. moshkovskii*) species, all subjects that were initially diagnosed as microscopically positive were not given any treatment at the moment. However, all the positive cases will be treated according to the Ministry of Health, Malaysia once the species have been identified.

## Results

### Study Cohort and Socioeconomic Profile

**Figure 2:**
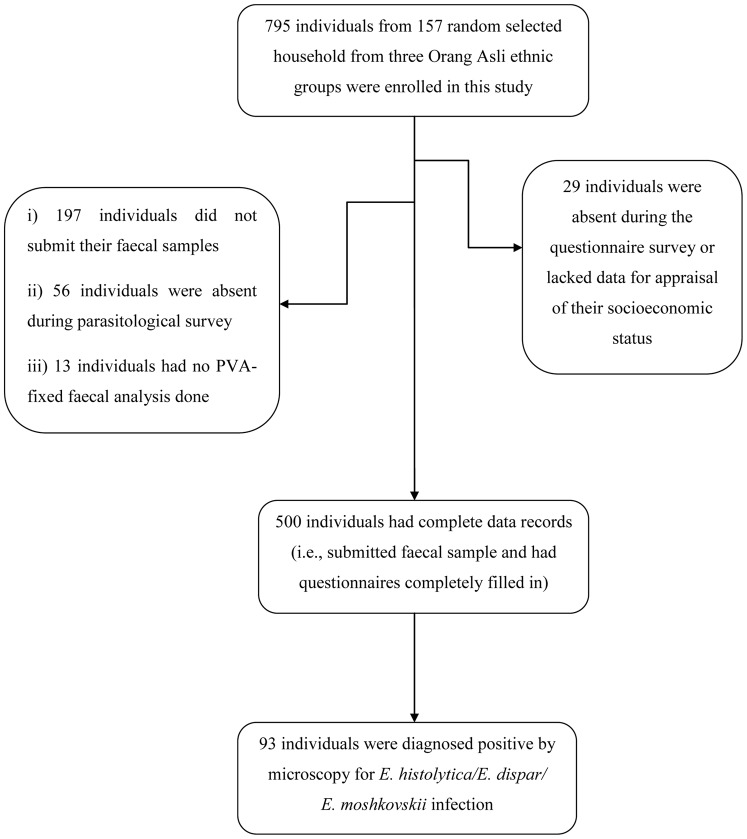
Study participants’ compliance of survey in three ethnic groups of Orang Asli.

From 795 enrolled participants, 253 individuals (31.8%) failed to submit their faecal samples and/or were absent during parasitological survey. Thirteen individuals (1.6%) had no PVA-fixed faecal sample and 29 individuals (3.7%) were absent during the household-based interviews, and hence their socioeconomic status could not be determined. Overall, 500 individuals (62.9%) were present during the cross-sectional study and respond to our questionnaire (Figure 2).

Among this cohort, 150 individuals (30%) were from Proto-Malay ethnic group, 139 (27.8%) from Negrito ethnic group, and 211 (42.2%) from Senoi ethnic group. The Proto-Malays comprised of 66 males (44%) and 84 females (56%) aged between 2 and 70 years old with a median age of 24 years [interquartile range (IQR) 10–39]. One hundred and thirty nine participants from the Negritos [66 males (47.5%); 73 females (52.5%)] aged between 2 and 74 years old with a median age of 13 years [interquartile range (IQR) 7–30], and 211 respondents among the Senois [87 males (41.2%); 124 females (58.8%)] aged between 2 and 70 years old with a median age of 20 years [interquartile range (IQR) 10–34] participated in this study. General characteristics of each ethnic group, including their socioeconomic profile are presented in Table 1.

**Table 1:** General characteristics of the Orang Asli communities that participated in this study.

Characteristics	Proto-Malay	Negrito	Senoi
	*n* (%)	*n* (%)	*n* (%)
**Overall**	150 (30.0)	139 (27.8)	211 (42.2)
**Age groups (years)**			
<15	59 (39.3)	72 (51.8)	90 (42.7)
15–24	17 (11.3)	22 (15.8)	34 (16.1)
25–44	48 (32.0)	29 (20.9)	54 (25.6)
45–74	26 (17.3)	16 (11.5)	33 (15.6)
**Gender**			
Male	66 (44.0)	66 (47.5)	87 (41.2)
Female	84 (56.0)	73 (52.5)	124 (58.8)
**Socioeconomic status**			
Father’s education (<6 years)	54 (66.7)	68 (77.3)	100 (76.3)
Mother’s education (<6 years)	53 (65.4)	77 (87.5)	102 (77.9)
Low monthly household income (<RM500)	17 (11.3)	115 (82.7)	128 (60.7)
Working mothers	45 (55.6)	33 (37.5)	75 (57.3)
Large family (≥8 members)	32 (21.3)	63 (45.3)	76 (36.0)
Supplied with piped water	143 (95.3)	76 (54.7)	138 (65.4)
Presence of toilet at household	143 (95.3)	48 (34.5)	117 (55.5)

*n* = Number examined. RM = Malaysian Ringgit; (US$1 = RM3.17) [16^th^ July 2012].

### Prevalence of *E. histolytica/E. dispar/E. moshkovskii*

The prevalence and distribution of *E. histolytica/E. dispar/E. moshkovskii* are shown in Table 2. The overall prevalence of this infection was 18.6%. It is evident that, of 211 subjects studied from the Senoi ethnic group, 18.5% were positive for *E. histolytica/E. dispar/E. moshkovskii*. However, subjects from the Negrito ethnic group had a relatively high prevalence of this infection which was 29.5%, whereby only 8.7% of the Proto-Malay ethnic group reported positive for *E. histolytica/E. dispar/E. moshkovskii*. Overall prevalence of *E. histolytica/E. dispar/E. moshkovskii* infection showed an age-dependency relationship, with significantly higher prevalence seen among participants aged less than 15 years old compared to those aged more than 15 years (χ^2^ = 4.237, *P* = 0.040). However, there was no significant difference of the *E. histolytica/E. dispar/E. moshkovskii* infection between males and females (χ^2^ = 2.433, *P* = 0.119). With regards to ethnic group, the prevalence of *E. histolytica/E. dispar/E. moshkovskii* infection was highest among those aged less than 15 years among the Proto-Malays (11.9%) and Senois (21.1%) while in Negritos, the prevalence was highest among the 45–74 age groups (37.5%).

**Table 2:** Prevalence of *E. histolytica/E. dispar/E. moshkovskii* among different Orang Asli ethnic groups according to age groups and gender.

	Overall			Proto-Malay			Negrito			Senoi		
	No. examined	No. infected	Prevalence (%)	No. examined	No. infected	Prevalence (%)	No. examined	No. infected	Prevalence (%)	No. examined	No. infected	Prevalence (%)
**Age groups (years)**												
<15	221	50	22.6	59	7	11.9	72	24	33.3	90	19	21.1
15–24	73	12	16.4	17	2	11.8	22	4	18.2	34	6	17.6
25–44	131	18	13.7	48	2	4.2	29	7	24.1	54	9	16.7
45–74	75	13	17.3	26	2	7.7	16	6	37.5	33	5	15.2
**Gender**												
Male	219	34	15.5	66	3	4.5	66	16	24.2	87	15	17.2
Female	281	59	21.0	84	10	11.9	73	25	34.2	124	24	19.4
**Total**	500	93	18.6	150	13	8.7	139	41	29.5	211	39	18.5

In addition, univariate analysis was also carried out based on different ethnic groups of Orang Asli and it was found that the Negrito ethnic group presented a greater risk of the infection than Proto-Malay ethnic group (Negrito *versus* Proto-Malay: χ^2^ = 20.602; *P*<0.001) and Senoi ethnic group (Negrito *versus* Senoi: χ^2^ = 5.764; *P* = 0.016).

### Parasite Associations

Table 3 summarizes significant associations between *E. histolytica/E. dispar/E. moshkovskii* infection with the demographic and lifestyle factors among different Orang Asli ethnic groups in Malaysia. Among the Proto-Malays, not washing hands after playing with soil or gardening (OR = 1.85; 95% CI = 1.02, 3.34; *P*<0.001), eating with hands (OR = 3.70; 95% CI = 1.03, 13.35; *P* = 0.004), and presence of other family members infected with *E. histolytica/E. dispar/E. moshkovskii* (OR = 1.76; 95% CI = 1.06, 2.92; *P*<0.001) showed significant association with *E. histolytica/E. dispar/E. moshkovskii* infection. As for the Negritos, four variables were found to be significantly associated with the infection; not washing hands after playing with soil or gardening (OR = 2.45; 95% CI = 1.20, 5.00; *P* = 0.005), eating with hands (OR = 2.43; 95% CI = 1.01, 5.83; *P* = 0.030), consuming raw vegetables (OR = 3.88; 95% CI = 1.02, 10.10; *P* = 0.026), and presence of other family members infected with *E. histolytica/E. dispar/E. moshkovskii* (OR = 2.06; 95% CI = 1.32, 3.22; *P*<0.001). Moreover, univariate analysis identified six factors that significantly associated with the infection among the Senois. These factors are drinking untreated water (OR = 2.04; 95% CI = 1.07, 3.88; *P* = 0.013), not washing hands after playing with soil or gardening (OR = 1.53; 95% CI = 1.06, 2.23; *P* = 0.007), close contact with domestic animals (OR = 3.35; 95% CI = 1.58, 7.12; *P*<0.001), eating with hands (OR = 3.06; 95% CI = 1.18, 7.95; *P* = 0.008), consuming raw vegetables (OR = 1.55; 95% CI = 1.11, 2.16; *P* = 0.001), and the presence of other family members infected with *E. histolytica/E. dispar/E. moshkovskii* (OR = 1.64; 95% CI = 1.18, 2.28; *P*<0.001). However, there was no significant association between *E. histolytica/E. dispar/E. moshkovskii* infection and diarrhoea or other gastrointestinal symptoms such as abdominal discomfort, vomiting, and nausea in all three ethnic groups.

**Table 3:** Associations between *E. histolytica/E. dispar/E. moshkovskii* infection and risk factors among different Orang Asli ethnic groups.

	Proto-Malay (*n* = 150)				Negrito (*n* = 139)				Senoi (*n* = 211)			
Variables	No. examined	%Infected	OR(95% CI)	*P*-value	No. examined	%Infected	OR(95% CI)	*P*-value	No. examined	%Infected	OR(95% CI)	*P*-value
**Age (years)**												
<15≥15	5991	11.96.6	1.34 (0.74, 2.45)1	0.262	7267	33.325.4	1.23 (0.82, 1.86)1	0.304	90121	21.116.5	1.15 (0.82, 1.59)1	0.396
**Gender**												
FemaleMale	8466	11.94.5	1.99 (0.73, 5.47)1	0.122	7366	34.224.2	1.31 (0.85, 2.01)1	0.197	12487	19.417.2	1.09 (0.71, 1.68)1	0.697
**Drinking untreated water**												
YesNo	9141	22.27.8	1.12 (0.89, 1.42)1	0.136	6970	36.222.9	1.41 (0.93, 2.15)1	0.084	13180	23.710.0	2.04 (1.07, 3.88)1	0.013^*^
**Bathing and washing in the river**												
YesNo	7143	14.38.4	1.04 (0.88, 1.22)1	0.588	6376	33.326.3	1.17 (0.82, 1.68)1	0.366	73138	23.315.9	1.20 (0.89, 1.61)1	0.191
**Not washing hands after playing with soil or gardening**												
YesNo	27123	25.94.9	1.85 (1.02, 3.34)1	<0.001^*^	9148	37.414.6	2.45 (1.20, 5.00)1	0.005^*^	79132	27.812.9	1.53 (1.06, 2.23)1	0.007^*^
**Close contact with domestic animals**												
NoYes	30120	13.37.5	1.17 (0.81, 1.70)1	0.310	6970	30.428.6	1.05 (0.72, 1.51)1	0.810	11992	28.66.5	3.35 (1.58, 7.12)1	<0.001^*^
**Indiscriminate defecation**												
YesNo	7143	14.38.4	1.04 (0.88, 1.22)1	0.588	9148	29.729.2	1.02 (0.61,1.68)1	0.951	94117	22.315.4	1.25 (0.87,1.79)1	0.196
**Sewage disposal**												
OutdoorCommon drainage	21129	19.07.0	1.27 (0.88, 1.83)1	0.068	10633	33.018.2	1.88 (0.84, 4.22)1	0.103	11398	22.114.3	1.36 (0.87, 2.13)1	0.143
**Eating with hands**												
YesNo	7080	15.72.5	3.70 (1.03, 13.35)1	0.004^*^	10534	34.314.7	2.43 (1.01, 5.83)1	0.030^*^	15358	22.96.9	3.06 (1.18, 7.95)1	0.008^*^
**Consuming raw vegetables**												
YesNo	6387	11.16.9	1.28 (0.70, 2.34)1	0.365	11326	33.611.5	3.88 (1.02, 10.10)1	0.026^*^	62149	32.312.8	1.55 (1.11, 2.16)1	0.001^*^
**Eating fresh fruits**												
YesNo	12921	9.34.8	1.90 (0.28, 13.03)1	0.493	12217	29.529.4	1.00 (0.38, 2.67)1	0.993	14467	20.813.4	1.46 (0.79, 2.69)1	0.197
**Father’s education**												
Non-educated (<6 yrs)Educated (>6 yrs)	5427	13.07.4	1.56 (0.44, 5.52)1	0.453	6820	32.425.0	1.33 (0.54, 3.28)1	0.531	10031	22.012.9	1.67 (0.64, 4.36)1	0.267
**Mother’s education**												
Non-educated (<6 yrs)Educated (>6 yrs)	5328	15.13.6	3.38 (0.52, 21.94)1	0.117	7711	33.89.1	4.43 (0.60, 32.87)1	0.097	10229	22.510.3	2.15 (0.70, 6.55)1	0.146
**Mother’s not working**												
YesNo	3645	13.98.9	1.28 (0.60, 2.73)1	0.477	5533	34.524.2	1.38 (0.72, 2.66)1	0.310	5675	28.614.7	1.51 (0.94, 2.44)1	0.052
**Household members**												
<8≥8	11832	10.23.1	2.94 (0.44, 19.83)1	0.209	7663	31.627.0	1.13 (0.74, 1.72)1	0.554	13576	18.518.4	1.00 (0.63, 1.60)1	0.986
**Household monthly income**												
≤RM500>RM500	17133	17.67.5	1.17 (0.86, 1.58)1	0.162	11524	31.320.8	1.59 (0.64, 3.97)1	0.306	12883	22.712.0	1.66 (0.94, 2.90)1	0.052
**Other family members infected with ** ***E.h/E.d/E.m***												
YesNo	13137	46.25.1	1.76 (1.06, 2.92)1	<0.001^*^	5683	48.216.9	2.06 (1.32, 3.22)1	<0.001^*^	55156	36.412.2	1.64 (1.18, 2.28)1	<0.001^*^
**Diarrhoea**												
YesNo	14136	14.38.1	1.03 (0.95, 1.12)1	0.433	11128	18.230.5	11.88 (0.43, 8.34)	0.391	22189	31.816.9	1.11 (0.95, 1.30)1	0.089
**Other symptoms of gastroenteritis**												
YesNo	7278	9.77.7	1.51 (0.92, 2.47)1	0.659	19120	15.831.7	12.23 (0.69, 7.25)	0.159	23188	21.718.1	1.03 (0.90, 1.17)1	0.670

RM  =  Malaysian Ringgit; (US$1 = RM3.17) [16^th^ July 2012. Reference group marked as OR  = 1 [OR  =  Odds Ratio]. CI  =  Confidence interval. ^*^Significant association (*P*<0.05).

### Risk Factors for *E. histolytica/E. dispar/E. moshkovskii* Infection

Table 4 shows the results from the logistic regression analysis between *E. histolytica/E. dispar/E. moshkovskii* infection and risk factors, taking into account the random effect of households. The data confirmed not washing hands after playing with soil or gardening and the presence of other family members infected with *E. histolytica/E. dispar/E. moshkovskii* as significant risk factors in all ethnic groups. It also further confirmed that eating with hands (OR = 3.65; 95% CI = 1.08, 12.34; *P* = 0.037), consuming raw vegetables (OR = 2.46; 95% CI = 1.08, 5.62; *P* = 0.032), and close contact with domestic animals (OR = 3.71; 95% CI = 1.31, 10.52; *P* = 0.014) were significant risk factors of *E. histolytica/E. dispar/E. moshkovskii* infection among the Senois.

**Table 4:** Multivariate analysis of risk factors associated with *E. histolytica/E. dispar/E. moshkovskii* infection among three ethnic groups of Orang Asli.

Variables	OR	95% CI	*P*-value
**(a) Proto-Malay**			
Not washing hands after playing with soil or gardening	6.21	1.52, 25.42	0.011
Presence of other family members infected with *E. histolytica/E. dispar/E. moshkovskii*	12.32	2.67, 56.99	0.001
**(b) Negrito**			
Not washing hands after playing with soil or gardening	3.89	1.46, 10.38	0.007
Presence of other family members infected with *E. histolytica/E. dispar/E. moshkovskii*	4.92	2.13, 11.36	<0.001
**(c) Senoi**			
Not washing hands after playing with soil or gardening	2.98	1.34, 6.60	0.007
Presence of other family members infected with *E. histolytica/E. dispar/E. moshkovskii*	2.62	1.14, 6.01	0.023
Eating with hands	3.65	1.08, 12.34	0.037
Consuming raw vegetables	2.46	1.08, 5.62	0.032
Close contact with domestic animals	3.71	1.31, 10.52	0.014

## Discussion

As shown by the results of the present study, *E. histolytica/E. dispar/E. moshkovskii* is still prevalent in Orang Asli communities in Malaysia with an overall prevalence of 18.6%. This prevalence rate is in agreement with previous studies conducted among Orang Asli communities (Senoi ethnic group) in Malaysia and reported a prevalence rate between 18.5% and 22.5% [12], [17]. On the other hand, previous study carried out in eight locations of Orang Asli communities which consist of Senoi and Proto-Malay ethnic groups reported a low prevalence rate (10.2%) of *E. histolytica/E. dispar/E. moshkovskii* infection [18]. However, the specific prevalence of *E. histolytica/E. dispar/E. moshkovskii* infection according to the location of the study area or different ethnic groups of Orang Asli was not reported in their study. In this present study, the prevalence rate of *E. histolytica/E. dispar/E. moshkovskii* infection according to the specific location of the study area was not reported as the three study locations have a similar climate which is a tropical rainforest climate, hot and humid, and has almost similar average rainfall and temperature throughout the year. Thus, it can be considered that the climate does not have any significant role in the distribution or dynamic transmission of this infection. Therefore, reporting the specific prevalence rate of this infection and it risk factors according to different ethnic groups are more meaningful for the public health authorities in the planning of specific measures to control *E. histolytica/E. dispar/E. moshkovskii* infection. This present study also observed some very encouraging trends. Individuals from the Negrito ethnic group have a greater risk of being infected by *E. histolytica/E. dispar/E. moshkovskii* as compared to those from the Senoi and Proto-Malay ethnic groups. This could be attributed to the housing condition and provision of basic amenities: most of houses in Proto-Malay villages have better housing condition and adequate provision of basic amenities as compared to Negrito and Senoi villages which still in traditional-built houses made up of wood or bamboo. Besides that, their behavioural habits in living beside a river is very crucial to them as most of their daily activities such as bathing, washing clothes, and household items are still carried out in the rivers. Nevertheless, some inhabitants preferred defecating at the site of the stream. Our findings are consistent with a previous study by Dunn [19] among 1,273 individuals from seven ethnic groups and sub-groups, the Negritos harboured more intestinal parasite species than any other ethnic group studied. Negritos who survive on hunting, fishing, and gathering do not significantly modify or simplify their habitat and therefore were subjected to a greater diversity of parasites compared to the other groups of aborigine living in surroundings that have been drastically simplified for settlement and cultivation. This proved that although sanitary behaviour appears to be similar for three ethnic groups, certain environmental and cultural practices and taboos may have interacted with their customary behaviour to produce different sanitary conditions [20]. However, this finding contradicted the reported prevalence of *Giardia intestinalis* infection carried out in the same communities which observed a high prevalence rate of giardiasis among members of the Proto-Malay ethnic group who have better housing condition and basic amenities [21]. It indicates that poor sanitary practices and poor provision of basic amenities play an important role in the transmission of *E. histolytica/E. dispar/E. moshkovskii* in the Negrito ethnic group.

Limitations of our study are as follows. First, the prevalence was based on a single faecal sample. Due to the intermittent nature of cyst excretion in the faeces, the prevalence rate is expected to be higher if three faecal samplings were collected and examined. Various studies of single faecal examinations have reported 10%–50% rate of false-negative results in examining the cysts and/or trophozoites [22], [23]. However, in our study it was not feasible to collect three faecal samples, due to the reasons stated in the materials and methods. Nonetheless, we have applied standard procedures during faecal collection and examination to overcome this limitation. Second, the method used for faecal analysis, formalin-ether sedimentation and trichrome staining, did not allow a distinction between *E. histolytica*, *E. dispar*, and *E. moshkovskii*. So, these parasites were indicated by *E. histolytica/E. dispar/E. moshkovskii*. More specialized methods now exist to distinguish them [24], [25] but remain inaccessible in the majority of developing countries [26].

It has been noted that those who do not washed their hands after playing with soil or gardening was at 2.98 to 6.21 higher risk of being infected with *E. histolytica/E. dispar/E. moshkovskii*. Moreover, we found that those eat with hands was 3.65 more likely of being infected. The major role of contaminated hands in the faecal-oral transmission of disease has been well documented in developing countries and washing hands before eating or after defecation has been considered as a secondary barrier. In Vietnam, the transmission routes via contaminated hands play a major role with a more than three folds risk increase if hands are not washed properly [27]. Previous study in Italy and Yemen showed that individuals who do not practice proper hand washing before eating are at two fold higher risk of getting *E. histolytica/E. dispar* infection [28], [29]. In addition, not washing hands has been reported to be significantly associated with diarrhoea in Malaysia [30], Myanmar [31], Bangladesh [32], and Indonesia [33].

Our findings suggest that *E. histolytica/E. dispar/E. moshkovskii* is highly infective within a family setting and these findings indicate the possibility of family members as the source of infection (carrier) and transmission occurred within family. Logistic regression analysis showed that individuals living with family members infected with *E. histolytica/E. dispar/E. moshkovskii* were at 2.62 (Senois), 4.92 (Negritos), and 12.32 (Proto-Malays) higher risk of being infected, respectively. Human-to-human contact within family members in all tribes is the possible mode of transmission particularly in situations where the frequency of transmission is high. In El Salvador, higher rates of infection have been observed in contacts of patients with amoebic liver abscess or with amoebic dysentery, or in asymptomatic carriers compared with controls [34]. A study in Mexico also found that 40% of contacts of *E. histolytica* and *E. dispar* carriers were also infected [35]. Under such circumstances, children may be at constant risk of infection as their parents might be the carrier of the cysts and this can be observed in this present study where the prevalence is high in children less than 15 years old. Screening of other family members should be recommended as one of the strategies in controlling *E. histolytica/E. dispar/E. moshkovskii* infection in all ethnic groups of Orang Asli community as an infected family member appears to be an important risk factor for this infection.

Another interesting finding of our study was the significant association observed between close contacts with domestic animal and *E. histolytica/E. dispar/E. moshkovskii* infection among Senoi ethnic group. From our observation, most Senois love to keep dog and cat as their household pets. These animals are left to wander freely in and out of the houses. Moreover, the cysts of *E.histolytica/E. dispar/E. moshkovskii* could be deposited on the surface (fur) of the animals during close contact with infected humans or from environment and then later transmitted to a next person. Study done by Wittnich [36] has reported *E. histolytica* infection in a German shepherd dog.

Epidemiologic studies have shown that consuming raw vegetables has two fold higher risk of acquiring *E. histolytica/E. dispar/E. moshkovskii* and this finding is consistent with a previous study from Brazil [37] and Iran [38]. By contrast, recent reports from Kenya, Yemen, Vietnam, and Tajikistan found no association between *E. histolytica* infection and the consumption of raw vegetables [27], [29], [39], [40]. We observed that tapioca shoots, wild fern shoots and locally planted leaves are the main raw vegetables as salad by the Senois; they usually do not wash these vegetables before eating. The significant association between consuming raw vegetables and *E. histolytica/E. dispar/E. moshkovskii* infection could be directly linked to the contamination with the cysts from the soil garden that vegetables were usually grown. Furthermore, it was frequently seen that vegetables were grown closely to the house where wastewater and human excreta were likely to be used often for irrigation and as fertilizers. Therefore, the current results highlight the potential of unwashed raw vegetables in transmission of *E. histolytica/E. dispar/E. moshkovskii* infection to Orang Asli communities. This study also emphasize the use of standard washing procedure (proper washing and disinfecting) before consumption of vegetables instead of traditional procedure (quick washing without disinfecting).

Finally, we found that there is no significant association between *E. histolytica/E. dispar/E. moshkovskii* infection and gastrointestinal symptoms including diarrhoea. It is well documented that 90% of *E. histolytica/E. dispar/E. moshkovskii* infected individuals are asymptomatic [41]. The possibility of harbouring the non-pathogenic species, *E. dispar* or *E. moshkovskii* cannot be ruled out. Moreover, it is now accepted that *E. dispar* infection is much more common than *E. histolytica* worldwide [42], [43]. Human infections with *E. moshkovskii* has also been reported in Tanzania, Bangladesh, India, Iran, Australia, and Turkey, and in general they are not associated with disease [44], [45], [46], [47], [48], [49]. However, further studies using molecular approaches are needed to distinguish the morphologically identical species of pathogenic and non-pathogenic species among different Orang Asli ethnic groups.

In conclusion, the present study showed high prevalence of *E. histolytica/E. dispar/E. moshkovskii* in Orang Asli communities with the highest prevalence was observed among Negrito ethnic group. Poor personal hygiene practices such as not washing hands after playing with soil or gardening and before eating and eating raw vegetables and close contact with domestic animals were the risk factors of *E. histolytica/E. dispar/E. moshkovskii* infection. Genotyping *E. histolytica/E. dispar/E. moshkovskii* from humans and animals are highly recommended to identify the species-specific and to understand the actual dynamics of transmission of these protozoa in Senoi ethnic group. Promoting awareness of good personal hygiene is of great importance to control this infection. Screening and giving treatment of the infected family members on the basis of one affected member would appear to be justified since human-to-human transmission is a common mode of acquisition of *E. histolytica/E. dispar/E. moshkovskii* infection among these communities. It is therefore vital for public health authorities to consider different planning and implementation of specific prevention and control strategies to reduce *E. histolytica/E. dispar/E. moshkovskii* infection significantly in different Orang Asli ethnic groups in Malaysia.

## References

[1] Walsh JA (1986) Problems in recognition and diagnosis of amoebiasis: estimation of the global magnitude of morbidity and mortality. Rev Infect Dis 8: 228–238. 2871619 10.1093/clinids/8.2.228

[2] World Health Organization (1997) World Health Organization/Pan American Health Organization/UNESCO report of a consultation of experts on amoebiasis. Wkly Epidemiol Rec 72: 97–100.9100475

[3] Hung CC, Ko NY, Ko WC, Lee HC, Ji DD, et al (2008) Amoebiasis among patrons visiting gay saunas in Taiwan. HIV Med 9: 787–789. 18624723 10.1111/j.1468-1293.2008.00609.x

[4] Haque R, Mondal D, Duggal P, Kabir M, Roy S, et al (2006) *Entamoeba histolytica* infection in children and protection from subsequent amoebiasis. Infect Immun 74: 904–909. 16428733 10.1128/IAI.74.2.904-909.2006 PMC1360358

[5] Tengku SA, Norhayati M (2011) Public health and clinical importance of amoebiasis in Malaysia: A Review. Trop Biomed 28: 194–222. 22041740

[6] Department of Orang Asli Development (JAKOA) (2011) Research and Development Division: Information Database, Jelebu District, Negeri Sembilan, Malaysia.

[7] Malaysia Meteorological Department (2012) Ministry of Science, Technology and Innovation Database, Malaysia.

[8] Department of Orang Asli Development (JAKOA) (2011) Research and Development Division: Information Database, Gerik District, Perak, Malaysia.

[9] Department of Orang Asli Development (JAKOA) (2011) Research and Development Division: Information Database, Temerloh District, Pahang, Malaysia.

[10] Kish L (1968) Survey sampling. John Wiley & Sons, Inc. New York, London.

[11] Nor Aza A, Ashley S, Albert J (2003) Parasitic infections in human communities living on the fringes of the Crocker Range Park Sabah, Malaysia. ASEAN *Review of Biodiversity and Environmental Conservation (ARBEC)*. January-March 2003.

[12] Hartini Y, Mohamed Kamel AG (2009) *Entamoeba histolytica/Entamoeba dispar* infection among Aborigines at Pos Lenjang, Pahang. Mal J Health Sc 7: 59–64.

[13] Lwanga SK, Lemeshow S (1991) Sample size determination in health studies: A Practical Manual. Geneva, Switzerland: World Health Organization.

[14] Salleh FM, Anuar TS, Yasin AM, Moktar N (2012) Wintergreen oil: A novel method in Wheatley’s trichrome staining technique. J Microbiol Methods 91: 174–178. 22986100 10.1016/j.mimet.2012.08.004

[15] Fleck SL, Moody AH (1993) Diagnostic technique in medical parasitology 11^th^ edition. Cambridge UP: 10–14.

[16] Markell EK, John DT, Krotoski WA (1999). Medical Parasitology (8^th^ ed). W.B Sunders Company, Philadelphia.

[17] Noor Azian MY, Lokman Hakim S, Maslawaty MN (2006) Use of molecular tools to distinguish *Entamoeba histolytica* and *Entamoeba dispar* infection among the aborigines in Cameron Highlands. Trop Biomed 23: 31–36. 17041549

[18] Ngui R, Ishak S, Chuen CS, Mahmud R, Lim YAL (2011) Prevalence and risk factors of intestinal parasitism in rural and remote west Malaysia. PLoS Negl Trop Dis 5: e974. 21390157 10.1371/journal.pntd.0000974 PMC3046966

[19] Dunn FL (1972) Intestinal parasitism in Malayan aborígenes (Orang Asli). Bull World Health Org 46: 99–113. 4537337 PMC2480635

[20] Che Ghani BM, Oothuman P (1991) Patterns of soil-transmitted helminth infection in relation to types of wáter supply, housing facilities and availability of latrines in rural area of Peninsular Malaysia. In: Collected papers on the control of soil-transmitted helmintiasis Vol. V: 64–71. APCO, Tokyo.

[21] Anuar TS, Al-Mekhlafi HM, Ghani MKA, Osman E, Yasin AM, et al (2012) Giardiasis among different tribes of Orang Asli in Malaysia: Highlighting presence of other family members infected with *Giardia intestinalis* as a main risk factors. Int J Parasitol 42: 871–880. 22846786 10.1016/j.ijpara.2012.07.003

[22] Marti H, Koella JC (1993) Multiple stool examinations for ova and parasites and rate of false-negative results. J Clin Microbiol 31: 3044–3045. 8263196 10.1128/jcm.31.11.3044-3045.1993 PMC266208

[23] Cartwright CP (1999) Utility of multiple-stool-specimen ova and parasite examinations in a high-prevalence setting. J Clin Microbiol 37: 2408–2411. 10405376 10.1128/jcm.37.8.2408-2411.1999 PMC85240

[24] Verweij JJ, Oostvogel F, Brienen EA, Nang-Beifubah A, Ziem J, et al (2003) Prevalence of *Entamoeba histolytica* and *Entamoeba dispar* in northern of Ghana. Trop Med Int Health 8: 1153–1156. 14641852 10.1046/j.1360-2276.2003.01145.x

[25] Abeba G, Amha K, Mekonnen M, Geremew T (2004) Detection and differentiation of two morphologically identical species of *Entamoeba* . Ethiop J Health Dev 18: 121–124.

[26] Okeke IN, Ojo O, Lamikanra A, Kaper JB (2003) Etiology of acute diarrhea in adults in southwestern Nigeria. J Clin Microbiol 41: 4525–4530. 14532177 10.1128/JCM.41.10.4525-4530.2003 PMC254369

[27] Pham Duc P, Nguyen-Viet H, Hattendorf J, Zinsstag J, Dan Cam P, et al (2011) Risk factors for *Entamoeba histolytica* infection in an agricultural community in Hanam province, Vietnam. Parasit Vectors 4: 102. 21663665 10.1186/1756-3305-4-102 PMC3141740

[28] Seppo R, Edgar JR, Regina GU, Nita G, Larry TG (2005) Prevalence and risk factors for protozoan and nematode infections among children in an Ecuadorian Highland community. Trans R Soc Trop Med Hyg 99: 585–592. 15916785 10.1016/j.trstmh.2005.01.003

[29] Naelah AA, Mohammed AKM, Rohela M, Yvonne LAL (2011) Factors associated with high prevalence of intestinal protozoa infections among patients in Sana’a City, Yemen. PLoS One 7: e22044. 10.1371/journal.pone.0022044 PMC3138770 21789210

[30] Knight S, Toodayan W, Caique W, Kyi W, Barnes A (1992) Risk factors for the transmission of diarrhoea in children: a case-control study in rural Malaysia. Int J Epidemiol 21: 812–818. 1521988 10.1093/ije/21.4.812

[31] Han A, Hlaing T (1989) Prevention of diarrhoea and dysentery by hand washing. Trans R Soc Trop Med Hyg 83: 128–131. 2603190 10.1016/0035-9203(89)90737-2

[32] Hoque BA, Chakraborty J, Chowdhury J, Chowdhury U, Ali M (1999) Effects of environmental factors on child survival in Bangladesh: a case-control study. Public health 113: 57–64. 10355303

[33] Gasem M, Dolmans W, Keuter M, Djokomoeljanto R (2001) Poor food hygiene and housing as risk factors for typhoid fever in Semarang, Indonesia. Trop Med Int Health 6: 484–490. 11422963 10.1046/j.1365-3156.2001.00734.x

[34] Spencer HC, Sullivan JJ, Mathews HM, Sauerbrey M, Bloch M, et al (1981) Serologic and parasitologic studies of *Entamoeba histolytica* in El Salvador. Am J Trop Med Hyg 30: 63–68. 6259960 10.4269/ajtmh.1981.30.63

[35] Ruiz-Palacios GM, Castanon G, Bojalil R, Tercero E, Raussel S, et al (1992) Low risk of invasive amebiasis in cyst carriers. A longitudinal molecular seroepidemiological study. Arch Med Res 23: 289–291. 1340317

[36] Wittnich C (1976) *Entamoeba histolytica* infection in a German shepherd dog. Can Vet J 17: 259–263. 184910 PMC1697359

[37] Benetton ML, Goncalves AV, Meneghini ME, Silva EF, Carneiro M (2005) Risk factors for infection by the *Entamoeba histolytica/Entamoeba dispar* complex: an epidemiological study conducted in outpatient clinics in the city of Manaus, Amazon Region, Brazil. Trans R Soc Trop Med Hyg 99: 532–540. 15869773 10.1016/j.trstmh.2004.11.015

[38] Shahnazi M, Jafari-Sabet M (2010) Prevalence of parasitic contamination of raw vegetables in villaje of Qazvin province, Iran. Foodborne Pathog Dis 7: 1025–1030. 20491596 10.1089/fpd.2009.0477

[39] Nyarango RM, Aloo PA, Kabiru EW, Nyanchongi BO (2008) The risk of pathogenic intetsinal parasite infections in Kisii Municipality, Kenya. BMC Public health 88: 237. 10.1186/1471-2458-8-237 PMC2478685 18620608

[40] Matthys B, Bobieva M, Karimova G, Mengliboeva Z, Jean-Richard V, et al (2011) Prevalence and risk factors of helminths and intestinal protozoa infections among children from primary schools in western Tajikistan. Parasit Vectors 4: 195. 21981979 10.1186/1756-3305-4-195 PMC3205355

[41] Stanley SL (2003) Amoebiasis. Lancet 361: 1025–1034. 12660071 10.1016/S0140-6736(03)12830-9

[42] Ramos F, Moran P, Gonzalez E, Garcia G, Ramiro M, et al (2005) High prevalence rate of *Entamoeba histolytica* asymptomatic infection in a rural Mexican community. Am J Trop Med Hyg 73: 87–91. 16014840

[43] Leiva B, Lebbad M, Winiecka-Krusnell J, Altamirano I, Tellez A, et al (2006) Overdiagnosis of *Entamoeba histolytica and Entamoeba dispar* in Nicaragua: a microscopic, Triage parasite panel and PCR study. Arch Med Res 37: 529–534. 16624654 10.1016/j.arcmed.2005.10.009

[44] Beck DL, Dogan N, Maro V, Sam NE, Shao J, et al (2008) High prevalence of *Entamoeba moshkovskii* in a Tanzanian HIV population. Acta Trop 107: 48–49. 18471796 10.1016/j.actatropica.2008.03.013 PMC2459240

[45] Haque R, Ali IKM, Clark CG, Petri Jr WA (1998) A case report of *Entamoeba moshkovskii* infection in a Bangladeshi child. Parasitol Int 47: 201–202.

[46] Parija SC, Khairnar K (2005) *Entamoeba moshkovskii* and *Entamoeba dispar* associated infection in Pondicherry, India. J Health Popul Nutr 23: 292–295. 16262027

[47] Solaymani-Mohammadi S, Rezaian M, Babaei Z, Rajabpour A, Meamar AR, et al (2006) Comparison of a stool antigen detection kit and PCR for diagnosis of *Entamoeba histolytica and Entamoeba dispar* infections in asymptomatic cyst passers in Iran. J Clin Microbiol 44: 2258–2261. 16757634 10.1128/JCM.00530-06 PMC1489450

[48] Fotedar R, Stark D, Marriott D, Ellis J, Harkness J (2008) *Entamoeba moshkovskii* infections in Sydney, Australia. Eur J Clin Microbiol Infect Dis 27: 133–137. 17957394 10.1007/s10096-007-0399-9

[49] Tanyuksel M, Ulukanligil M, Guclu Z, Araz E, Koru O, et al (2007) Two cases of rarely recognized infection with *Entamoeba moshkovskii.* . Am J Trop Med Hyg 76: 723–724. 17426178

